# Supplementary material for: T2N as a new tool for robust electrophysiological modeling demonstrated for mature and adult-born dentate granule cells
Source: eLife. 2017 Nov 22;6:e26517. doi: 10.7554/eLife.26517 (PMC5737656; doi:10.7554/eLife.26517)
Supplement: Supplementary file 1. [file elife-26517-supp1.docx]

**Supplementary File 1: Electrophysiology in mature mouse GCs – experiment vs. classical GC model.**

| **Intrinsic properties** | **Experiment** | **Classical model reconstructed morphologies** | **Classical model synthetic morphologies** |
| --- | --- | --- | --- |
| R_in_ [MΩ] (@ -82.1mV) | 289.5 ± 34.9 | 406.9 ± 19.1 | 406.6 ± 9.6 |
| c_m_ [pF] | 48.9 ± 5.3 | 77.6 ± 4.6 | 78.3 ± 2.1 |
| tau [ms] | 34.0 ± 2.0 | 37.5 ± 0.3 | 37.7 ± 0.1 |
| V_rest_ [mV] | -92.7 ± 0.5* | -71.2 ± 0.2 | -71.1 ± 0.1 |
| I_threshold_ [pA] | 47.5 ± 4.5 | 46.9 ± 3.0 | 44.7 ± 1.2 |
| V_threshold_ [mV] | -46.3 ± 1.6 | -41.2 ± 0.6 | -40.9 ± 0.1 |
| AP amplitude [mV] | 95.6 ± 2.1 | 77.9 ± 1.4 | 72.0 ± 0.7 |
| AP width [ms] | 1.03 ± 0.02 | 4.42 ± 0.20 | 4.44 ± 0.05 |
| fAHP [mV] | 15.7 ± 1.4 | -** | -** |
| Interspike interval [ms] | 36.3 ± 4.9 | 25.0 ± 11.4 | 46.4 ± 10.4 |
| Max. spike slope [V/s] | 450.1 ± 23.7 | 268.5 ± 22.5 | 283.7 ± 7.3 |
| gKir [nS] | 5.46 ± 1.31 | 0.17 ± 0.09*** | 0.21 ± 0.05*** |

- after subtraction of a calculated liquid junction potential of 12.1 mV.

** the first AP did not show an fAHP as defined in Materials and Methods

*** no Kir channel exist in the Aradi & Holmes (1999) model, the low amount of open channels at hyperpolarized potentials probably originates from the included A-type K-channel
